# Unmasking chronic bone cement pulmonary embolism: acute hypoxia two years post-vertebroplasty

**DOI:** 10.36416/1806-3756/e20260058

**Published:** 2026-09-14

**Authors:** Guoping Yang, Weiwei Liao

**Affiliations:** 1. Department of Joint Surgery, Ganzhou Hospital-Nanfang Hospital, Southern Medical University (Ganzhou People’s Hospital), Ganzhou, Jiangxi, China.; 2. Medical Imaging Center, Ganzhou Hospital-Nanfang Hospital, Southern Medical University (Ganzhou People’s Hospital), Ganzhou, Jiangxi, China.

A 68-year-old woman presented with a 20-day history of intermittent chest tightness and dyspnea. Laboratory tests showed elevated cholesterol (5.52 mmol/L) and triglycerides (1.73 mmol/L); pulse oximetry was 93%. Cardiac enzymes and coronary angiography were normal. Notably, she underwent percutaneous vertebroplasty (PVP) two years prior. Non-contrast CT revealed chronic T5 and T6 compression fractures with visible bone cement traces (Figure 1A), a dilated pulmonary trunk (diameter ~34.5 mm), a linear high-density opacity in the right main pulmonary artery, and multiple linear and patchy opacities in both lungs (Figure 1B). Subsequent CT angiography confirmed that these lesions were located within the pulmonary artery lumens and small peripheral branches (Figures 1C and 1D), establishing the diagnosis of bone cement pulmonary embolism (BCPE) associated with pulmonary hypertension. 

**Figure 1:**
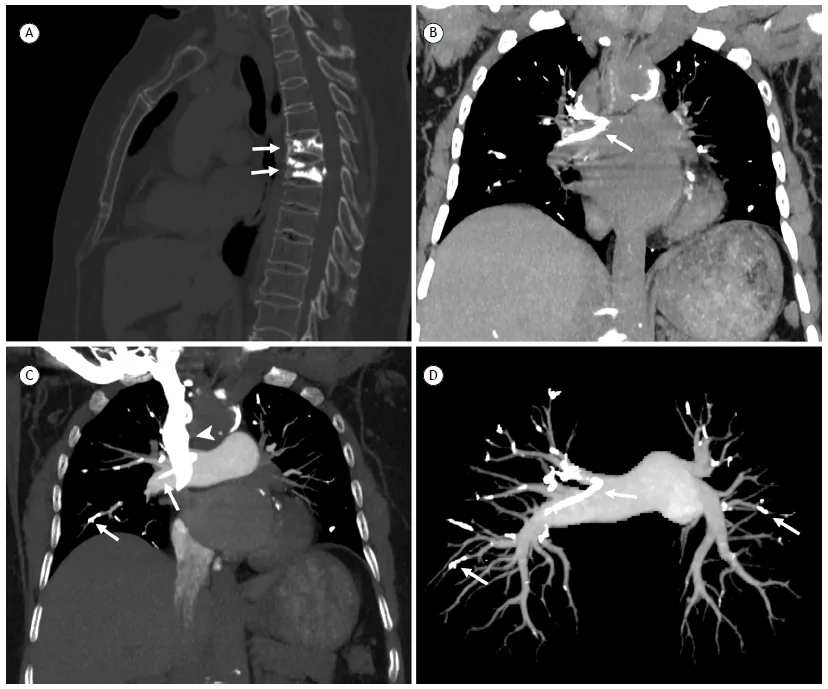
Imaging findings in a 68-year-old woman two years post-vertebroplasty: In A, sagittal CT reconstruction in the bone window reveals chronic T5 and T6 compression fractures with visible bone cement traces (arrow); in B, coronal non-contrast chest CT shows thick linear high-density opacities (arrow) at the origin of the right main pulmonary artery and its upper and lower lobe branches, representing bone cement emboli, alongside multiple small scattered lesions throughout both lungs; in C and D, coronal CT angiography reconstruction and maximum intensity projection images clearly demonstrate these emboli localized within the lumens of the right main pulmonary artery and small peripheral branches (arrows).

Following oxygen therapy and anticoagulation-and after declining endovascular thrombectomy-her symptoms resolved, and she remained asymptomatic at the 3-month follow-up. 

BCPE has a reported incidence ranging from 0% to 25%.^1^ Because most cases are asymptomatic, they often go undetected or are discovered only at a later stage, sometimes never being recognized during a patient’s lifetime; conversely, reported symptomatic cases typically manifest as acute events shortly after the procedure.^2^ Consequently, long-term surveillance for BCPE is often neglected. However, persistent pulmonary hypertension caused by cement emboli can gradually impair cardiopulmonary function.^3^ Furthermore, embolic instability may trigger late-onset acute symptoms. This case underscores BCPE as a latent post-PVP threat, emphasizing the need for timely imaging evaluation to ensure early detection and continued monitoring.
